# The score of integrated disease surveillance and response adequacy (SIA): a pragmatic score for comparing weekly reported diseases based on a systematic review

**DOI:** 10.1186/s12889-019-6954-3

**Published:** 2019-05-22

**Authors:** Bien-Aimé Makasa Mandja, Didier Bompangue, Pascal Handschumacher, Jean-Paul Gonzalez, Gérard Salem, Jean-Jacques Muyembe, Frédéric Mauny

**Affiliations:** 1 Service de Microbiologie, Faculté de Médecine, Université de Kinshasa, Kinshasa, Democratic Republic of Congo; 2 Laboratoire Chrono-Environnement, UMR 6249 CNRS, Université de Bourgogne Franche-Comté, Besançon, France; 3 UMR 912 SESSTIM, INSERM, IRD, U2, Université d’Aix-Marseille, Strasbourg, France; 4 Department of Microbiology and Immunology, Division of Biomedical Graduate Research Organization, Georgetown University School of Medicine, 4000 Reservoir Road, Washington, D.C., NW 20057 USA; 5 Université Paris Ouest Nanterre, Paris, France; 6 Institut National de Recherche Biomédicale, Kinshasa, Democratic Republic of Congo; 7 Centre Hospitalier Universitaire de Besançon, uMETh, Besançon, France

**Keywords:** Adequacy score, Democratic Republic of Congo, Infectious diseases, Integrated disease surveillance and response, Reported data

## Abstract

**Background:**

The Integrated Disease Surveillance and Response (IDSR) strategy implemented by the World Health Organization (WHO) in Africa has produced a large amount of data on participating countries, and in particular on the Democratic Republic of Congo (DRC). These data are increasingly considered as unevaluable and, therefore, as requiring a rigorous process of validation before they can be used for research or public health purposes. The aim of this study was to propose a method to assess the level of adequacy of IDSR morbidity data in reflecting actual morbidity.

**Methods:**

A systematic search of English- and French-language articles was performed in Scopus, Medline, Science Direct, Springer Link, Cochrane, Cairn, Persée, and Erudit databases. Other types of documents were identified through manual searches. Selected articles focused on the determinants of the discrepancies (differences) between reported morbidity and actual morbidity. An adequacy score was constructed using some of the identified determinants. This score was applied to the 15 weekly reported diseases monitored by IDSR surveillance in the DRC. A classification was established using the Jenks method and a sensitivity analysis was performed. Twenty-three classes of determinants were identified in 35 IDSR technical guides and reports of outbreak investigations and in 71 out of 2254 researched articles. For each of the 15 weekly reported diseases, the SIA was composed of 12 items grouped in 6 dimensions.

**Results:**

The SIA classified the 15 weekly reported diseases into 3 categories or types: high score or good adequacy (value > = 14), moderate score or fair adequacy (value > = 8 and < 14), and low score or low or non-adequacy (value < 8). Regardless of the criteria used in the sensitivity analysis, there was no notable variation in SIA values or categories for any of the 15 weekly reported diseases.

**Conclusion:**

In a context of sparse health information in low- and middle-income countries, this study developed a score to help classify IDSR morbidity data as usable, usable after adjustment, or unusable. This score can serve to prioritize, optimize, and interpret data analyses for epidemiological research or public health purposes.

**Electronic supplementary material:**

The online version of this article (10.1186/s12889-019-6954-3) contains supplementary material, which is available to authorized users.

## Introduction

Sub-Saharan Africa has the highest total morbidity and mortality burden of infectious diseases in the world [1]. In 2012, global morbidity in the region was estimated at 74,000 per 100,000 inhabitants. This number is nearly twice that of the Eastern Mediterranean or South-East Asian regions (40,779 and 40,341 per 100,000 inhabitants, respectively) [2]. In addition, approximately half the mortality from all types of infectious diseases in the world occurs in Sub-Saharan Africa [3, 4]. While emerging non-transmittable diseases have expanded in the region in the last two decades, infectious diseases remain a major public health concern [3, 4]. By the end of the 1990s, most sub-Saharan African countries had seen major public health events or outbreaks. Huge epidemics occurred in the DRC, including the large cholera outbreak in the Rwandan Hutu refugee camp in Goma in 1994 [5], the 1000 cases of poliomyelitis reported in Mbuji-Mayi in 1995 [6, 7], and the largest ever monkey pox outbreak that took place in Sankuru in 1996–1997 [8]. Overall, the growing epidemic risk in sub-Saharan African countries is a source of major international concern.

In 1998, an Integrated Disease Surveillance and Response (IDSR) strategy was initiated under the guidance of the WHO Regional Office for Africa (WHO AFRO) to prevent and control these multiple epidemic emergencies [9, 10]. This strategy works by reinforcing national public health surveillance and response systems in the region. According to the IDSR technical guidelines, the specific objectives of the strategy are to: 1) integrate vertical disease surveillance systems for effective and efficient use of resources; 2) improve the flow and use of information for detecting and responding to public health threats; and 3) improve country capacity to detect and respond to priority public health events [10, 11]. The IDSR strategy, which relies on systematic and continuous data collection and reporting by health care facilities, has eight functions: identification, notification, analysis and interpretation, epidemic investigation and confirmation, preparation, response, circulation of information and evaluation, and improvement of the system [11–14]. Depending on the health specificity of each country, WHO AFRO recommends IDSR surveillance of a number of priority transmittable diseases (weekly reporting) and non-transmittable diseases (monthly reporting) [11]. This timely continuous epidemiological surveillance improves the availability and use of data on the leading causes of illness, death, and disability in the region [15]. As such, the IDSR strategy contributes to high-level decision-making in the area of public health in participating countries.

In addition to contributing to disease outbreak prevention and control, IDSR morbidity data are increasingly relevant to epidemiological research. In particular, they are now being used to determine the spatial and temporal dynamics of various diseases [16–18]. They thus constitute an alternative to other techniques for generating health data in low and middle-income countries (e.g., demographic health surveys, STEPS surveys, household surveys, etc.). While these techniques can produce reliable data, they are indeed very costly to implement [19]. In the DRC, more and more epidemiological studies rely on the large amount of data that have been produced since the 1999 implementation of the IDSR strategy.

It has been demonstrated, however, that the IDSR strategy results in social and spatial discrepancies (differences) in disease distribution between reported cases (reported morbidity) and field reality (actual morbidity). In the particular context of the DRC, this is likely because IDSR morbidity data reporting is based on a syndromic approach [19–22]. Unfortunately, these discrepancies cast doubt on the validity of epidemiological studies using IDSR morbidity data. Because the true value and accuracy of these data are difficult to evaluate [19, 20, 22], there is a pressing need to develop a method for validating them before they can be used for research or public health purposes.

While some approaches have been proposed to assess the quality of IDSR morbidity data, they focus on a limited number of diseases. These approaches include: 1) a method for assessing the “relevance and validity” of IDSR morbidity data on chickenpox, hepatoma, anaemia, malnutrition, and measles [20]; 2) a method for constructing, comparing and spatializing selected indicators and indices for analyses of malaria based on IDSR morbidity data [19]; or 3) a method using a tree model scenario to estimate the proportion of lost cases of monkey pox in the DRC health system due to IDSR data reporting [23]. To our knowledge, no method applicable to all diseases monitored by IDSR surveillance and assessing the discrepancies between “reported morbidity” and “actual morbidity” has been published.

The aim of this article is to propose a method for assessing the level of adequacy of IDSR morbidity data in reflecting the actual morbidity of the 15 weekly reported diseases monitored by IDSR surveillance in the DRC.

## Methods

### Study setting

**Fig. 1:**
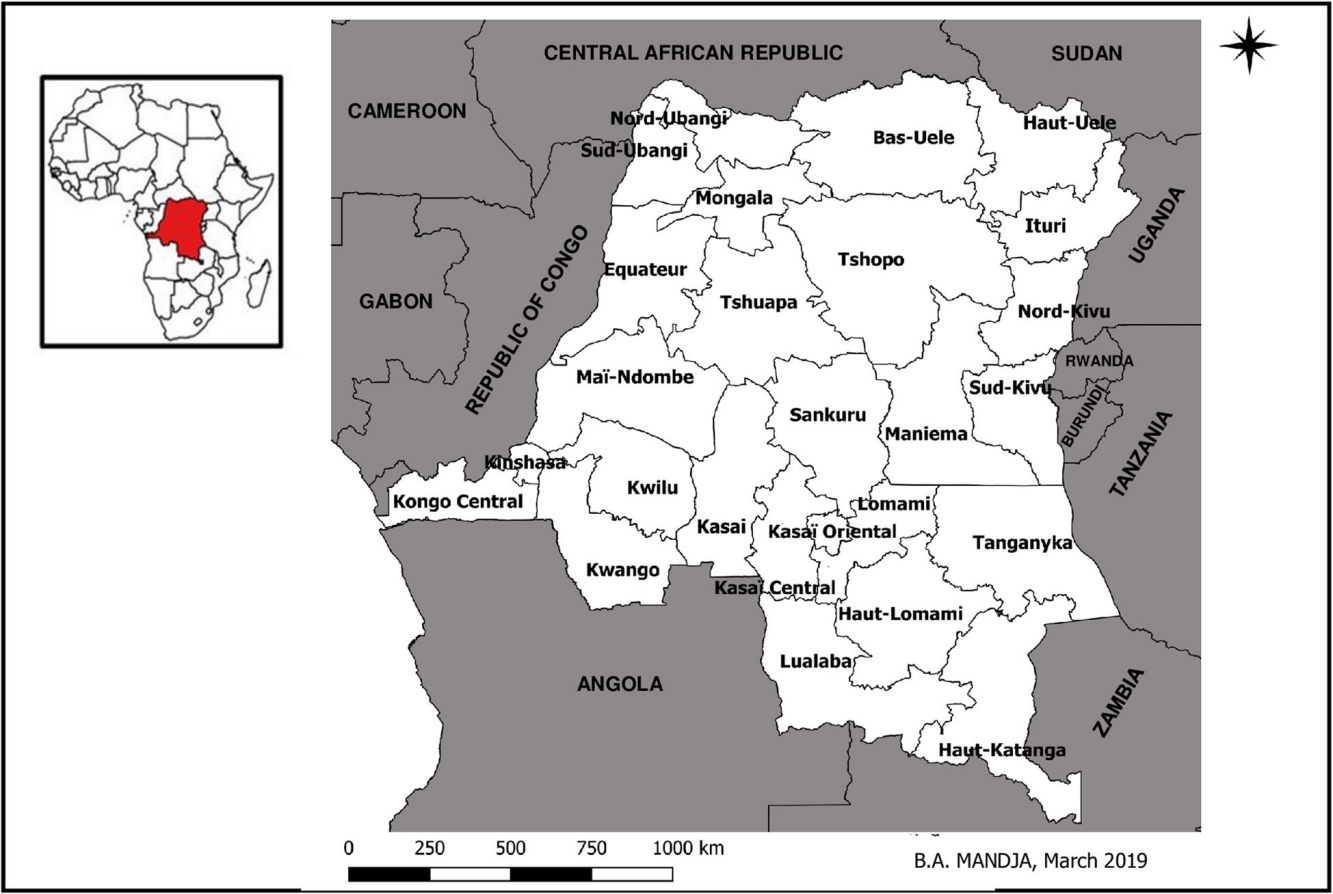
Administrative map of the DRC including 26 new provinces and bordering countries, (Source: The map was created with the provincial Shapefile obtained from the free, open, collaborative platform Common geographical reference of DRC (https://data.humdata.org/dataset/dr-congo-settlements). The map was created using the free software QGIS 12.8 geographical information system)

Located in Central Africa, the DRC had a total area of 2.3 million km^2^ and an estimated population of 86,895,208 inhabitants in 2016 [24]. In 2015, the country was subdivided into 26 administrative provinces (Fig. 1). The health system in the DRC is a three-tier (central, intermediate, and peripheral) pyramidal structure. The central level sets standards and is composed of the Minister’s office and the general secretariat, which includes 13 directorates and 52 specialized programs. The intermediate level has a technical and logistical role and is composed of 26 provincial health divisions and provincial hospitals. The peripheral level has an operational role in the implementation of primary health care. This level consists of 515 Health Zones (HZ), which include 393 General Reference Hospitals (GRH) and 8504 planned Health Areas (HA), 8266 of which have a Health Center (HC) [25].

### Organization of the surveillance system in the DRC

**Fig. 2:**
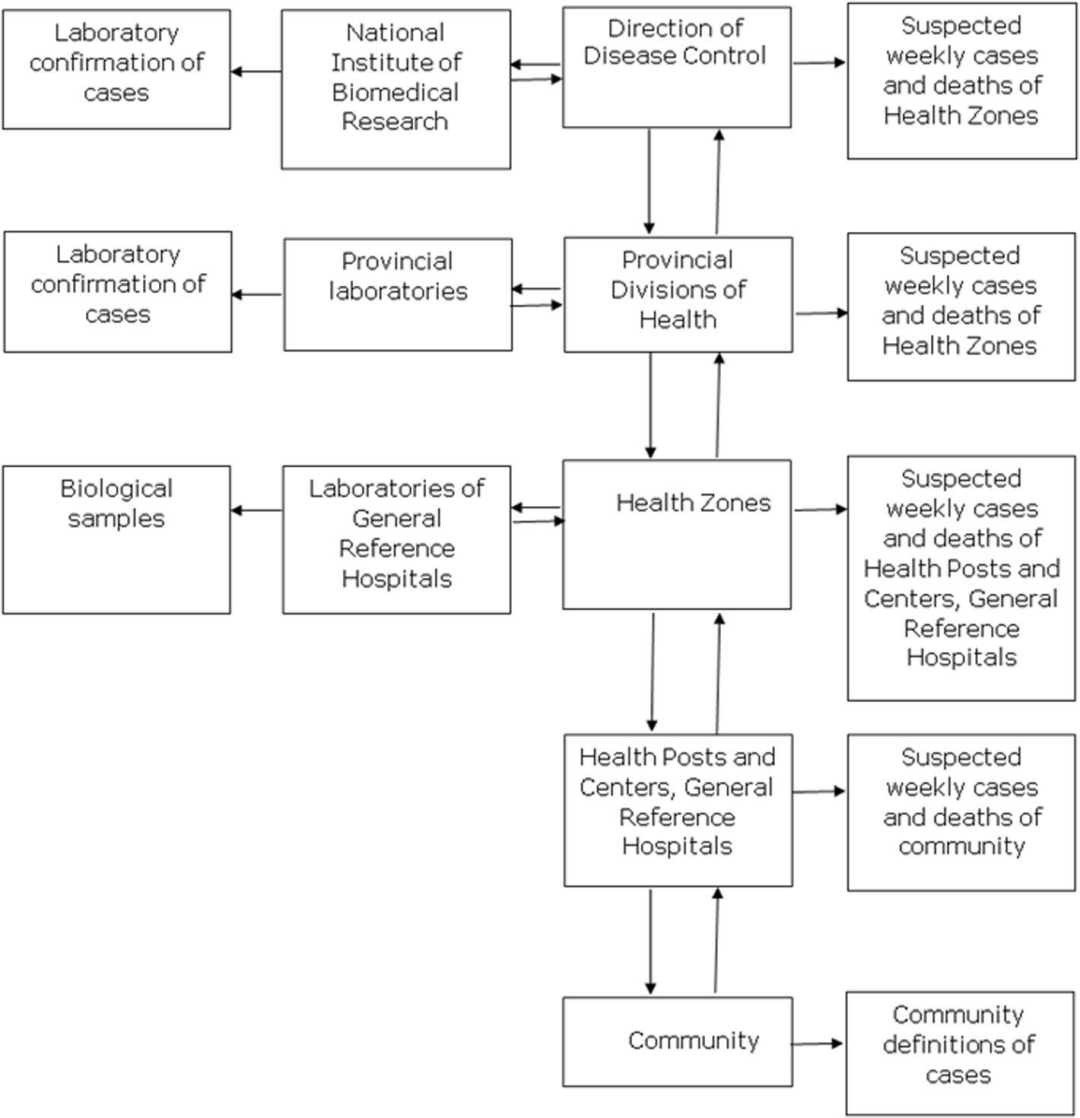
Flow chart representing the organization of the surveillance system in the DRC

In the DRC, the IDSR strategy is managed by the General Direction of Disease Control (GDDC). Since 2000, the DRC has been monitoring 12 weekly reported diseases with acute epidemic potential, namely: acute flaccid paralysis, bloody diarrhoea, cholera, haemorrhagic fevers, malaria, measles, meningitis, monkeypox, neonatal tetanus, pertussis, plague, and yellow fever. In 2010, acute respiratory infections, rabies, and typhoid fever were added to the list of weekly reported diseases. In 2016, dracunculiasis and maternal deaths were also included on the list. The DRC also organises the monthly reporting of 20 endemic and priority health problems. Suspected cases are identified using the WHO clinical case definitions (syndromic approach). Cases are diagnosed and recorded on hard copies by nurses in health centers and by Medical Officers in General Reference Hospitals. The Medical and the Clinical Officers of the private sector are also integrated in IDSR and participate in identification and notification of priority diseases in HZs. Data are reported electronically from the different HZs to the provincial health divisions, and then centralized in the GDDC (Fig. 2). The quality of the data is checked at each level during weekly epidemiological meetings [10, 11].

### Construction and calculation of the score of IDSR adequacy

A specific score, the “Score of IDSR Adequacy (SIA),” was designed to assess the level of adequacy of IDSR morbidity data. The level of adequacy of IDSR morbidity data is the ability of these data to reflect real or exact morbidity. No individual clinical data were used to construct the SIA, only bibliographical data. The score was applied to the 15 weekly reported diseases in the DRC, and was constructed according to the following procedures:

- 1) Literature review focusing on the discrepancies between reported morbidity and actual morbidity; 2) Identification of the determinants of the discrepancies between reported morbidity and actual morbidity; 3) Selection of items to be included in the score from the determinants identified in the literature; 4) Construction of the theoretical score; 5) Application of the constructed score to the 15 weekly reported diseases monitored by IDSR surveillance in the DRC; 6) Classification of the 15 diseases using the constructed score; and 7) Performance of a sensitivity analysis on the constructed score. Steps 1 and 2 were conducted using PRISMA guidelines [26]; the PRISMA checklist of items is summarized in Additional file 1: Appendix 1.

### 1) Literature review focusing on the discrepancies between reported morbidity and actual morbidity

#### Data sources and search strategy

The search was performed in 7 peer-reviewed literature databases: Embase, Medline, Web of Science, Cochrane, Scopus, Cairn.info and Persée, from their inception to December 2016. The keywords in French were: *surveillance épidémiologique, données administratives, informations sanitaires*, *statistiques sanitaires, morbidité rapportée, morbidité* r*éelle et qualité de données*. The keywords in English were: *epidemiological surveillance, administrative data, health information, health statistics, reported morbidity, real morbidity and data quality*. Queries combining the above keywords were performed using the Boolean search operators “AND” and “OR” to identify the most relevant articles. The full search strategy per database is included in Supporting information in Additional file 2: Appendix 2. Technical guides and reports (DRC Ministry of Health, WHO offices, Epicentre) were also included in our review through manual searches.

### Eligibility criteria

The selected articles focused on: 1) the determinants of the discrepancies between reported morbidity and actual morbidity; and 2) the epidemiological surveillance of infectious diseases in low- and middle-income countries.

Only English- and French-language articles were included in the study.

### Study selection

Two independent reviewers screened the articles on the basis of title and abstract. They then selected the articles that matched the eligibility criteria.

### 2) Identification of the determinants of the discrepancies between reported morbidity and actual morbidity

#### Data extraction process and data items

Two independent reviewers used a standardized questionnaire to extract the following bibliographical data in duplicate: name of authors, year of publication, country of study, and determinants of the discrepancies between reported morbidity and actual morbidity. Disagreements between reviewers were solved by consensus.

### Synthesis of results

The determinants of the discrepancies (differences) between “reported morbidity” and “actual morbidity” were defined as major factors that can induce distortions between actual morbidity and health information gathered by health care facilities (reported morbidity). These distortions can occur at different stages of patient care. Three main stages can be distinguished: 1) people’s perception of illness and health care; 2) diagnosis by health care providers; and 3) data reporting to the national IDSR database. Based on the literature review, 23 classes of determinants were identified (see Table 1). These classes of determinants are presented in Additional file 3: Table S1.

**Table 1:** Classes of determinants of discrepancies of reported morbidity to actual morbidity

stages of patient care	Classes of determinants or potential distorting factors	References
Perception of the illness and Health care access	Form and perceived severity of illness	[35–40]
	Etiological concept and type of disease	[38, 40–47]
	Individual characteristics of patients	[38, 43, 48–55]
	Socio-economic characteristics of patients	[38, 43, 54–65]
	Attractiveness factors of places	[66–71]
	Geographical accessibility	[38, 42–44, 61, 72–75]
	Characteristics of the environment	[66, 76–78]
	Urbanization	[79–83]
	Movements of population	[84]
Diagnosis	Presence of an intervention program	[22, 85, 86]
	Functioning of health services: quality of service and framework	[38, 44, 48, 58, 62, 87–90]
	Staff competence	[44, 62, 87, 91]
	Lack of supplementary diagnosis	[44, 92]
	Lack of clinical standardized decision tree for diagnosing	[91, 93, 94]
	Difficulty of differential diagnosis with other diseases	[20, 31–34]
	Spatial distribution of disease	[95–97]
Data reporting	Staff competence	[84]
	Standardization of data collection tools	[84, 98]
	Large volume of work	[31]
	Falsification of data	[31]
	Typing errors	[31, 99]
	Integration of the disease into a national or global strategy	[10, 100]
	Characteristic of the response	[10, 100]

### 3) Selection of items to be included in the score from the determinants identified in the literature

To construct the Score of IDSR Adequacy (SIA), we selected determinants with the following 4 characteristics: availability, ability to discriminate, sensitivity, and reproducibility. Availability is the ability of the determinant to be collected easily. Ability to discriminate is the ability of the determinant to represent relatively homogeneous sub-groups. Sensitivity is the ability of the values of the determinant to change if the situation changes. Reproducibility is the ability of the values of the determinant not to change if the situation does not change. Of the determinants detailed in Additional file: Table S1, 12 were selected; these 12 determinants are presented in Table 2. In the SIA score, the selected determinants were named items and the classes of determinants were named dimensions according the score terminology.

**Table 2:** Score of Integrated Disease Surveillance and Response adequacy (SIA), defined for the present study including: dimensions, items and codes

Dimension	Item	Code ^#^
Form & perceived severity of illness*	Incubation period	0 = > 14 days; 1 = 7–14 days; 2 = < 7 days
	Onset of disease	0 = Mild; 2 = Severe with symptoms
	Symptoms in the acute phase	0 = Mild; 2 = Severe
	Contagiousness	0 = Low; 1 = Moderate; 2 = High
	Death rate (%) without Treatment	0 = < 5; 1 = 6–19; 2 = > = 20
Etiological concept & disease type**	Disease Local Name	0 = Without; 1 = Changing according localities; 2 = Shared by localities
Differential diagnosis with other diseases**	Number of epidemics reported each year during the last five years	0 = Less than 2; 1 = 2 to 4; 2 = 5
	PPV***	0 = < 20%; 1 = 20–50%; 2 = > = 50%
Spatial distribution of disease**	Proportion of health zones affected by epidemics of each of the 15 diseases (%).	0 = less than 20%; 1 = more than 20%
Integration of the disease into a national or global strategy**	Internationally funded research	0 = Without; 1 = With
	National or International eradication programs	0 = Not; 1 = yes
Response Characteristic*	Timely Response	0 = delayed response; 1 = Immediate

PPV = Positive predictive value

^a^ # = Value Numbers

^b^ * = Criteria intrinsically related to pathology

^c^ ** = Criteria changing between countries and over time

### 4) Construction of the theoretical score

The response to each SIA item was coded as 0/1, 0/2, or 0/1/2. These different code weights were assigned to the SIA items to account for the relative influence of people’s perception of illness and health care (36). The highest code values (2 or 1 for items coded as 0/1) were attributed to the items that facilitate people’s perception of illness and health care, diagnosis by health care providers, and/or data reporting. For each disease, the SIA was the sum of the code values for all weighted items. The theoretical SIA ranged from 0 to 20 points (Table 2). The coding of each SIA item for cholera is shown in Additional file 4: Appendix 3.

### 5) Application of the constructed score to the 15 weekly reported diseases monitored by IDSR surveillance in the DRC

The SIA was calculated for the 15 weekly reported diseases (acute flaccid paralysis, acute respiratory infections, bloody diarrhoea, cholera, haemorrhagic fevers, malaria, measles, meningitis, monkeypox, neonatal tetanus, pertussis, plague, rabies, typhoid fever, and yellow fever) [14]. We focused on these diseases because they were the only ones to be monitored by IDSR surveillance in the DRC prior to 2015. The distribution of observed responses to each item (codes) was described. Redundancy of items in each dimension was assessed using the Kappa coefficient.

### 6) Classification of the 15 diseases using the constructed score

The score was discretized using both the Jenks method and the natural thresholds method. The Jenks method provided the most homogeneous categories using an iterative procedure that allowed for minimizing intra-class variance and for maximizing inter-class variance. It was the most suitable for discretizing the overall score for each disease. The natural thresholds method categorized the 15 diseases taking into account the discontinuities of the series. [27, 28]. It was performed as a comparative method to confirm the robustness of our analysis. These two discretization procedures allowed for assessing the quality of the data produced for each of the 15 weekly reported diseases according to their calculated score.

The Jenks method and the natural thresholds methods were selected because they help to constitute homogenous categories. In our study, they allowed for classifying diseases into 3 categories or types: Types I, II, and III. Type I is composed of diseases with a score greater than or equal to 14, (high score). This score indicates good adequacy of IDSR morbidity data, meaning that the data can be used for epidemiological research or public health purposes. Type II is composed of diseases with a score ranging from 8 to 14 (moderate score). This score indicates fair adequacy of IDSR morbidity data, meaning that the data can be used after adjustment for epidemiological research or public health purposes. Type III is composed of diseases with a score smaller than 8 (low score). This score indicates low or non-adequacy of IDSR morbidity data, meaning that the data cannot be used for epidemiological research or public health purposes.

### 7) Performance of a sensitivity analysis on the constructed score

To check the robustness of the score, a sensitivity analysis was performed in two ways: 1) by iteratively removing one item at a time; and 2) by modifying the numerical values of the item codes (values 0, 1, 2 changed to 0, 1, to 0, 2, 4, etc.). Removing one item at a time allowed us to assess the potential major effect of each item on the overall score, and modifying the item code values allowed us to assess the effects of the code weights. After each modification, the score was recalculated, and the newly obtained score was discretized using the Jenks method. For each of the 15 diseases, the new score rankings and categories were compared to the initial score rankings and categories to ensure that no significant variation had occurred.

### Characteristics of the selected studies

**Fig. 3:**
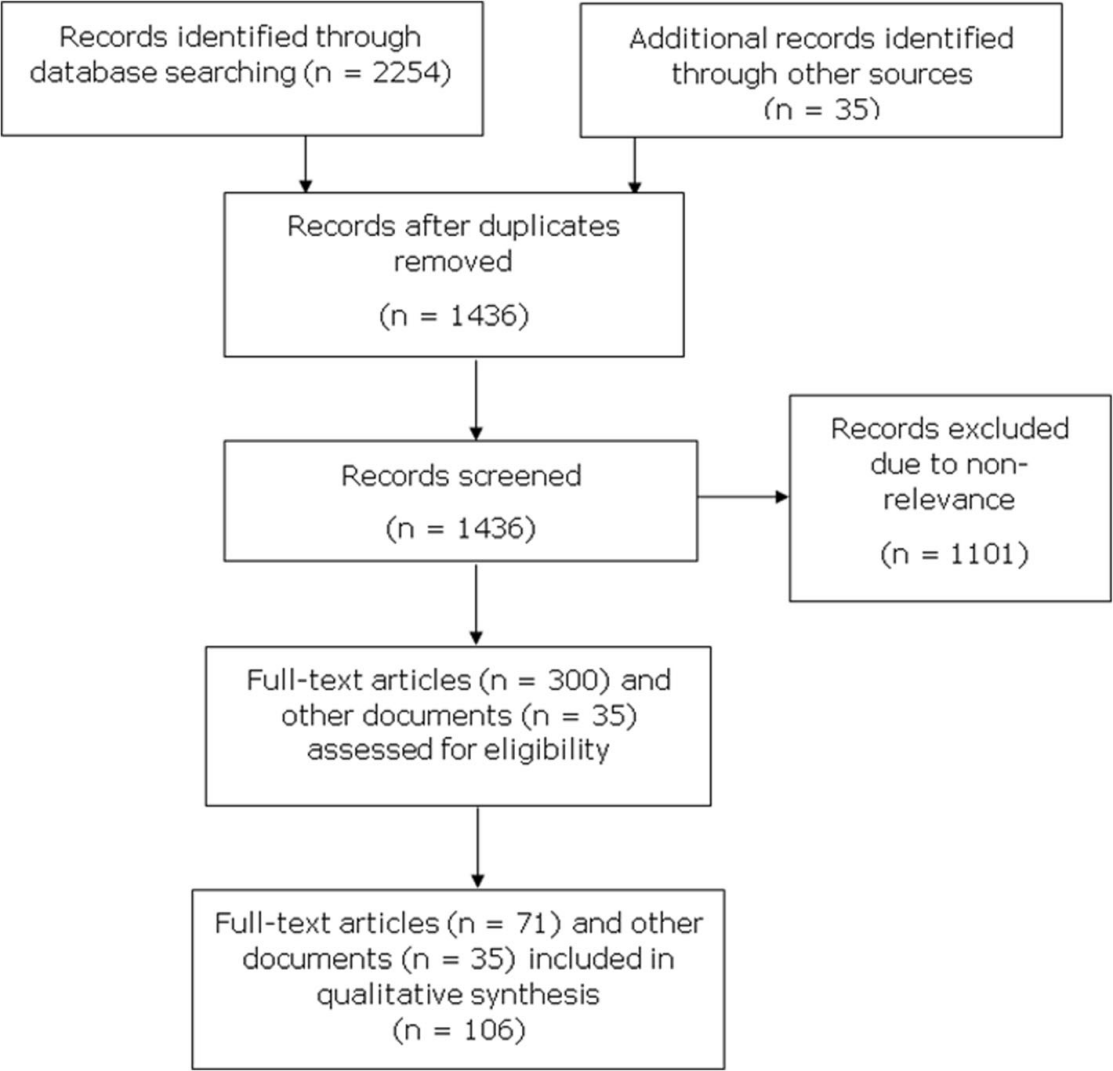
PRISMA flow diagram of the article selection process

The protocol search strategy yielded 2254 abstracts. Of these, 853 duplicate records were removed. The 1401 remaining abstracts were screened, and 1101 were excluded due to non-relevance. Of the 300 remaining articles with full texts, 71 matched the inclusion criteria (Fig. 1). The 71 included articles were published between the years 1974 and 2014. Of these, 41 articles focused on infectious diseases and 54 on low- and middle-income countries; 45 were written in English; and 44 were published after the year 2000 (date of initiation of IDSR in Africa). Five IDSR technical guides [11–14] and 30 technical reports (*n* = 30) were also included in our bibliographical research (Fig. 3). These documents provided crucial information on the context and factors favouring the onset of outbreaks, the operational case definitions, the local names of diseases, the types of intervention, the number of epidemics reported each year by DRC region, the biological confirmation of cases, and the modalities of epidemic response.

### Synthesis of results

Twelve of the identified determinants were selected to be used as SIA items. These 12 items were related to the six dimensions of the SIA (one to 5 items per dimension) (Table 2). Six items are linked to people’s perception of illness and health care: incubation period, onset of disease, symptoms in the acute phase, contagiousness, death rate (%) without treatment, and local disease name. Three items are linked to diagnosis: number of epidemics reported each year during the last five years, positive predictive value (PPV), and proportion of health zones affected by epidemics of each of the 15 diseases (%). The last three items are linked to data reporting: internationally funded research, national or international eradication programs, and timely response.

## Results

When the SIA was applied to the 15 weekly reported diseases, SIA values ranged from 4 (rabies) to 19 points (cholera). The mean score was 10.7 points (Table 3). Agreement between items in each dimension ranged from 0.03 to 0.47 (Kappa coefficients). All numerical code values proposed for each item were used at least once.

**Table 3:** Calculation of the score of IDSR adequacy among 15 weekly reported diseases by the Integrated Disease Surveillance and Response (IDSR) WHO program implemented in the Democratic Republic of Congo

Disease	F&P Severity			ECT			DD		Spatial D	Strategy		Response	Total	%
	IP	OD	SAP	Cont	DRT	DLN	NER	PPV	PAE	IFR	NIEP	TR		
Cholera	2*	2	2	2	2	2	2	1	1	1	1	1	19	95
Measles	1	0	2	2	2	2	2	1	1	1	1	1	16	80
BD	2	2	2	1	1	1	1	1	1	1	0	1	14	70
HF	1	2	2	2	2	1	0	2	0	1	0	1	14	70
NT	2	0	2	0	2	1	0	2	1	1	1	0	12	60
MPX	1	0	2	2	1	1	2	2	0	1	0	0	12	60
AFP	0	0	2	2	1	1	0	1	1	1	1	1	11	55
Pertussis	1	0	2	2	1	1	0	1	1	1	0	0	10	50
Plague	2	0	0	2	2	1	1	1	0	0	0	1	10	50
MNG	2	0	0	2	2	0	1	0	1	1	0	1	10	50
ARI	2	0	0	1	2	1	1	1	1	0	0	0	9	45
YF	2	0	2	2	2	0	0	0	0	0	0	0	8	40
Malaria	1	0	0	0	2	1	0	1	1	1	0	0	7	35
TF	1	0	0	1	2	0	0	0	1	0	0	0	5	25
Rabies	0	0	0	0	2	1	0	0	1	0	0	0	4	20

*AFP* Acute flaccid paralysis, *ARI* Acute respiratory infections, *BD* Bloody diarrhoea, *Cont*., Contagiousness, *DD* Differential Diagnosis, *DLN* Disease Local Name, *DRT* Death rate (%) without Treatment, *ECT* Etiological concept and type of disease, *F&P* Severity, Form and perceived severity, *HF* Hemorrhagic fevers, *IFR* Internationally funded research, *IP* Incubation period, *MNG* Meningitis, *MPX* Monkeypox, *NER* Number of epidemics reported each year during the last five years, *NIEP* National/International eradication programs, *NT* Neonatal tetanus, *OD* Onset disease, *PAE* Proportion of health zones affected by epidemics of each of the 15 diseases (%), *PPV* Positive predictive value, Response, Response Characteristic, *SAP* symptoms in the acute phase, Spatial D, Spatial distribution of disease, Strategy, Disease & national or global strategy, Response, Response Characteristic, *TF* Typhoid fever, *TR* Timely Response, *YF* Yellow fever

^a^ * All values in the colons refer to the Table 2

**Fig. 4:**
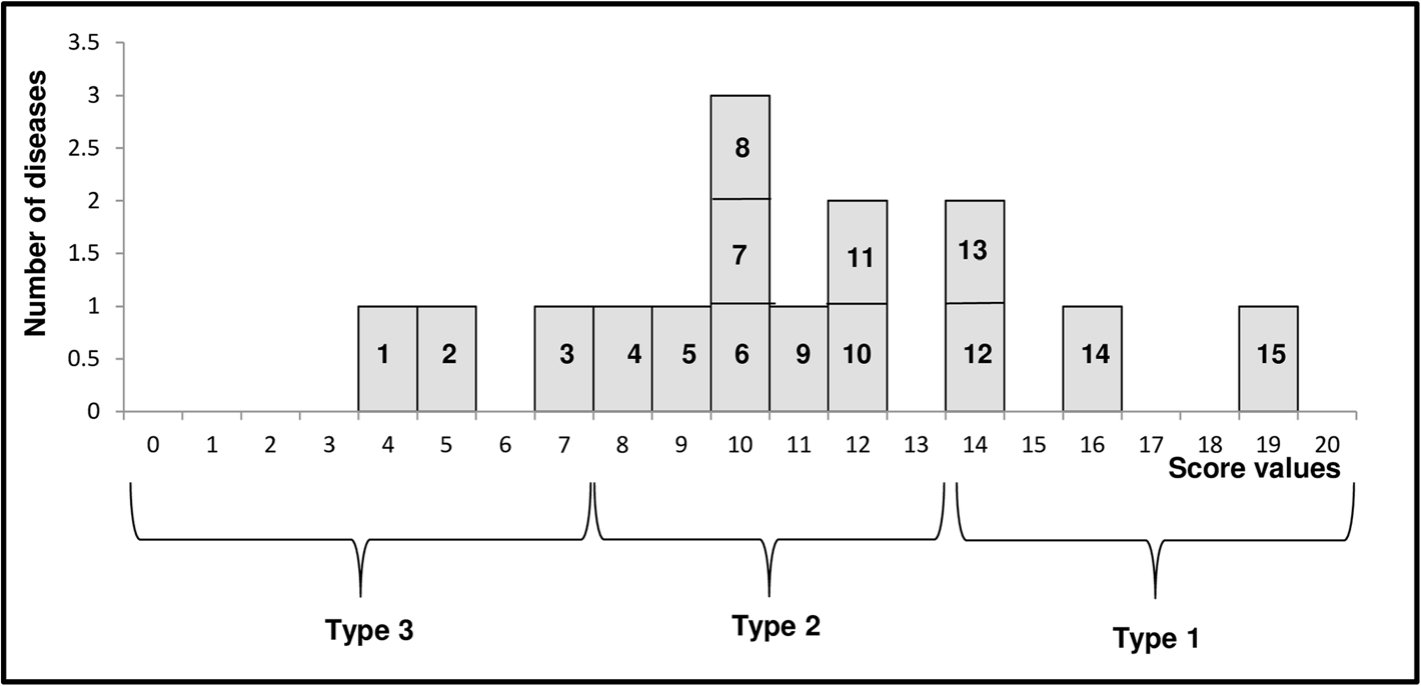
Classification of the 15 weekly reported diseases monitored by IDSR surveillance in the DRC (according to the Jenks method), ^a^Type 1, high score (value > = 14): hemorrhagic fevers (12); bloody diarrhea (13); measles (14); cholera (15). ^b^Type 2, moderate score (value > = 8 and < 14): yellow fever (4); acute respiratory infections (5); meningitis (6); plague (8); pertussis (8); acute flaccid paralysis (9); monkeypox (10); neonatal tetanus (11). ^c^Type 3, low score (value < 8): rabies (1); typhoid fever (2); malaria (3).^d^ Discretization using the natural thresholds method yielded almost the same classification, except in the case of malaria, which was classified as Type 2 instead of Type 3

After discretization, 3 categories of diseases were identified as follows (Fig. 4):

- Type 1: High score (value > = 14) (good adequacy: IDSR morbidity data can be used for epidemiological research or public health purposes); this category included cholera, measles, haemorrhagic fevers and bloody diarrhoea;
- Type 2: Moderate score (value > = 8 and < 14) (fair adequacy: IDSR morbidity data can be used after adjustment for epidemiological research or public health purposes); this category included neonatal tetanus, monkeypox, acute flaccid paralysis pertussis, meningitis, acute respiratory infections and yellow fever;
- Type 3: Low score: (value < 8) (low or non-adequacy: IDSR morbidity data cannot be used for epidemiological research or public health purposes); this category included malaria, typhoid fever and rabies.

### Additional analysis

The results of the sensitivity analysis, both by iteratively removing one item at a time and by modifying the item code values, are presented in Tables 4 and 5. Iteratively removing one item at a time did not lead to changes in the score ranking, as the highest-ranking diseases were still cholera and measles (19 and 16, respectively) and the lowest-ranking ones were still yellow fever, malaria, typhoid fever, and rabies (8, 7, 5, and 4, respectively). Modifying the item code values led to minor changes in the score ranking of the other pathologies (differences of no more than one or 2 ranks). However, the same 3 categories resulted from discretization. Minor changes were observed when the following items were iteratively removed: “incubation period,” “onset of disease,” and “symptoms in the acute phase.” No changes were observed when the other items were removed, and the distribution of the diseases into the 3 categories was only slightly modified.

**Table 4:** Sensitivity analysis of score of IDSR adequacy by removing iteratively one item at a time of calculated codes of the 15 weekly reported diseases by the IDSR in DRC

Ty.	Disease	Score	Removed item **											
			IP	OD	SAP	Cont	DRT	DLN	NER	PPV	PAE	IFR	NIEP	TR
I	Cholera	19*	17	17	17	17	17	17	17	18	18	18	18	18
	Measles	16	15	16	14	14	14	14	14	15	15	15	15	15
	BD	14	**13** ^**#**^	**12** ^**#**^	**12** ^**#**^	13	13	13	14	13	14	13	14	13
	HF	14	**12** ^**#**^	**12** ^**#**^	**12** ^**#**^	12	12	13	13	**12** ^**#**^	13	13	14	13
II	NT	12	11	12	10	12	11	11	12	10	12	11	12	12
	MPX	12	**10^**	12	10	10	10	11	**10^**	10	11	11	11	12
	AFP	11	**11^**	11	**9^**	9	10	10	**11^**	10	10	10	10	10
	Pertussis	10	9	10	**10^**	8	9	10	10	10	10	10	10	10
	Plague	10	**8** ^**#**^	10	**10^**	8	8	9	9	9	9	9	10	9
	MNG	10	**8** ^**#**^	10	**8^**	8	8	9	9	9	9	9	10	9
	ARI	9	**7** ^**#**^	9	**9^**	8	7	8	8	8	8	9	9	9
	YF	8	**6** ^**#**^	**8** ^**#**^	**6^**	**6**^	**6** ^**#**^	8	8	8	8	8	8	8
III	Malaria	7	6	7	**7^**	**7**^	5	6	7	6	6	6	7	**7** ^**#**^
	TF	5	4	5	5	4	3	5	5	5	4	5	5	5
	Rabies	4	4	4	4	4	2	3	4	4	3	4	4	4

AFP Acute flaccid paralysis, ARI Acute respiratory infections, BD Bloody diarrhoea, Cont. Contagiousness, DLN Disease Local Name, DRT Death rate (%) without Treatment, HF Hemorrhagic fevers, IFR internationally funded research, IP Incubation period, MNG Meningitis, MPX Monkeypox, NER Number of epidemics reported each year during the last five years, NIEP National/International eradication programs, NT Neonatal tetanus, OD Onset disease, PAE Proportion of health zones affected by epidemics of each of the 15 diseases (%), PPV Positive predictive value, SAP symptoms in the acute phase, Response Response Characteristic, TF Typhoid fever, TR Timely Response, YF Yellow fever

^a^ * All values in the colons refer to the Table 3

^b^ ** see Table 2

^c^ Type I: SIA > = 14; Type II: SIA = 8–13; Type III: SIA < 8

^d **^**^, Number in bold denote SIA value that changes the rank of the disease in the same type

^e **#**^, Number in bold denote SIA value that changes the type of disease

**Table 5:** Sensitivity analysis of score of IDSR adequacy by modifying the numerical values of the item calculated codes of the 15 weekly reported diseases by the IDSR in DRC

Ty.	Disease	Score	Modifying the numerical values of the item codes **				
			CC1	CC2	CC3	CC4	CC5
I	Cholera	19*	17	23	27	12	32
	Measles	16	15	20	24	11	29
	BD	14	**12** ^**#**^	**17** ^**#**^	**20** ^**#**^	**9**^	**22** ^**#**^
	HF	14	**12** ^**#**^	**16** ^**#**^	**18** ^**#**^	**11**^	**22** ^**#**^
II	NT	12	11	15	**20**^	8	21
	MPX	12	11	**13**^	18	8	21
	AFP	11	10	15	14	**9**^	19
	Pertussis	10	10	13	**18**^	8	**20**^
	Plague	10	10	12	**17**^	7	**20**^
	MNG	10	9	11	14	7	17
	ARI	9	9	**10** ^**#**^	**16**^	7	**18**^
	YF	8	**7** ^**#**^	**8** ^**#**^	**13** ^**#**^	**4** ^**#**^	**13** ^**#**^
III	Malaria	7	7	**9**^	11	**6**^	**14**^
	TF	5	5	6	9	4	10
	Rabies	4	4	5	7	3	8

AFP Acute flaccid paralysis, ARI Acute respiratory infections, BD Bloody diarrhoea, Cont. Contagiousness, DLN Disease Local Name, DRT Death rate (%) without Treatment, HF Hemorrhagic fevers, IFR internationally funded research, IP Incubation period, MNG Meningitis, MPX Monkeypox, NER Number of epidemics reported each year during the last five years, NIEP National/International eradication programs, NT Neonatal tetanus, OD Onset disease, PAE Proportion of health zones affected by epidemics of each of the 15 diseases (%), PPV Positive predictive value, SAP symptoms in the acute phase, Response Response Characteristic, TF Typhoid fever, TR Timely Response, YF Yellow fever

^a^ * All values in the colons refer to the Table 3

^b^ ** see Table 2

^c^ Type I: SIA > = 14; Type II: SIA = 8–13; Type III: SIA < 8

^d **^**^, Number in bold denote SIA value that changes the rank of the disease in the same type

^e **#**^, Number in bold denote SIA value that changes the type of disease

## Discussion

The application of the SIA to the 15 weekly reported diseases monitored by IDSR surveillance in the DRC yielded 3 categories or types: high score or good adequacy (value > = 14; usable data), moderate score or fair adequacy (value > = 8 and < 14; usable data after adjustment), and low score or low or non-adequacy (value < 8; non-usable data).

Overall, our examination of studies from a wide range of disciplines, as well as of IDSR technical guides and reports of outbreak investigations, gave a strong foundation to this study. The SIA is the outcome of sound interdisciplinary work, combining the expertise of specialists in the fields of integrative health geography, public health, epidemiology, infectious diseases, and medical anthropology. It also draws on 20 years of experience and feedback from field practitioners responsible for care structures in rural areas of the DRC.

The SIA was constructed using a pragmatic and robust assessment method. The agreement between items varied from low to moderate, indicating a low redundancy of items in each dimension. All the code values assigned to SIA items were used at least once. The sensitivity analysis confirmed the robustness of the score: neither the score ranking nor the classification of the different diseases were modified when iteratively removing one item at a time, or when modifying the item code values. However, for some Type-2 diseases, the score ranking was slightly modified, even as the classification remained unchanged.

According to the PRISMA checklist, there should be no bias within or across studies included in systematic reviews. Our systematic review was not concerned by this requirement because no meta-analysis was included. Only qualitative studies were screened for determinants that can induce discrepancies between reported morbidity and actual morbidity.

Other methods have been proposed to study the adequacy of reported morbidity in reflecting actual morbidity. However, these methods focused on a single disease or on a limited number of diseases [19, 20, 23]. To our knowledge, the SIA is the first method that can apply to all 15 weekly reported diseases monitored by IDSR surveillance in the DRC. As such, it allows for the extensive assessment of the quality of data collected on a wide range of diseases, including neglected diseases that are not integrated into major global strategies (like pertussis or plague).

Our study found coherence in the value of the score obtained for each of the diseases. The SIA confirms the low quality of data produced on diseases (such as malaria) monitored using syndromic surveillance [22, 29]. Moreover, it allows for analysing data on certain pathologies (neonatal tetanus, acute respiratory infections, and rabies) that are less frequently investigated. Unlike previous attempts at assessing data quality (usually data on a single health problem or disease) with tools using binary variables (presence/absence, positive/negative, or yes/no) [20], the SIA can quantify the adequacy of data in reflecting the actual morbidity of each monitored disease. It is therefore one of the only tools available to pragmatically assess the quality of IDSR morbidity data. This can be of great relevance to the various users and actors involved in the implementation of surveillance and response strategies like the IDSR.

Nevertheless, this study has limitations that must be acknowledged. Our literature review may have missed some relevant determinants that could have been included in the score. However, only currently available and accessible information in the DRC were used to construct the SIA. It is therefore unlikely that an additional determinant would have affected the consistency and validity of the score.

Another limitation of our study is that the SIA was applied to weekly reported diseases that are monitored using the syndromic approach. The code values of 3 items (number of epidemics reported each year during the last 5 years; PPV; and proportion of health zones affected by epidemics of each of the 15 diseases) would likely change if the data on these diseases concerned biologically confirmed cases. It is therefore also likely that the score ranking of the different diseases would change if the SIA were applied to diseases not monitored using the syndromic approach.

In our study, cholera had the highest score among all Type-1 diseases. The good adequacy of IDSR cholera data in reflecting actual cholera morbidity has already been demonstrated in a study by Bompangue et al. This study examined the spatial and temporal dynamic of cholera outbreaks using IDSR morbidity data: it identified lacustrine sanctuary areas as the site of emergence of all cholera outbreaks in the DRC [16, 17]. These findings have led to significant advances in the understanding of the mechanisms involved in the spread and recurrence of cholera outbreaks. They have also prompted the DRC Ministry of Health to adjust the national strategy for cholera control by targeting cholera sanctuary areas [18]. The success of this adjusted strategy may be seen as a retroactive validation of the quality of IDSR data on this disease. This is in line with our findings, whereby IDSR data on cholera have a high level of adequacy in reflecting actual cholera morbidity, as calculated using the SIA.

Similarly, the SIA classification of measles as a Type-1 disease corroborates the findings of a study by Fasin et al. [20]. Of the five diseases or health conditions analysed in this study (chickenpox, hepatoma, anaemia, malnutrition and measles), only data on measles morbidity had a good level of adequacy in reflecting actual measles morbidity. This pathology was also the only one to show both satisfactory relevance and quality [20]. These findings are unsurprising because in low and middle-income countries, where measles prevalence remains high, health workers are well-equipped to recognize and produce a clinical diagnosis of the disease and diagnosis is oriented in the absence of prior vaccination [30].

Adjusted data on monkeypox, a Type-2 disease as per the SIA, have been used in a study conducted in the DRC by Hoff et al. This study using IDSR morbidity data on monkeypox proposed two adjustment methods: a comparison of monkey pox with two control diseases (acute flaccid paralysis and neonatal tetanus), and a scenario tree model to estimate the proportion of potentially lost cases in the DRC health system [23].

Our study indicates that IDSR morbidity data on yellow fever, malaria, typhoid fever, and rabies (Type-3 diseases) should not be used for epidemiological research or public health purposes, due to their low level of adequacy in reflecting actual morbidity. Until 2014, the clinical and presumptive diagnosis of malaria based solely on fever likely induced many false positives. Several infectious diseases can cause fever, which may explain the large gap between supposed and actual cases of malaria [22, 29, 31]. The same applies to yellow fever and typhoid fever, which can be confused with several other pathologies when they are syndromically diagnosed. One of the main reasons for the misdiagnosis of rabies, and for the limited use of data on the disease, may be its long incubation period. It is indeed difficult for health workers to link a dog bite that occurred 1 to 3 months earlier to recent symptoms. The clinical diagnosis of rabies can also be difficult if the specific signs of hydrophobia or aerophobia are not present [32, 33]. Lastly, bat-acquired cases of rabies may be missed because health care providers fail to inquire into the history of bat bites [34].

## Conclusion

Our study found that the quality of IDSR morbidity data is highly variable from one disease to another. If confirmed, these findings could prompt a revision of the IDSR strategy as it is applied in low- and middle-income countries. The different algorithms of disease surveillance could be reconsidered and improved, especially those that rely on a syndromic approach. Lastly, the SIA could be applied: 1) to monthly reported diseases; and 2) to weekly reported diseases in other low- and middle-income countries that are not monitored using a syndromic approach.

## Additional file

Additional file 1: Appendix 1. PRISMA 2009 Checklist. A checklist includes 27 items deemed essential for transparent reporting of a systematic review. (DOCX 32 kb). (DOCX 31 kb)

Additional file 2: Appendix 2. . Database search strategies. Description of data: Appendix 2 includes the search terms for the epidemiological surveillance, administrative data, health information, health statistics, reported morbidity, real morbidity and data quality. (DOCX 35 kb)

Additional file 3: **Table S1**. Classes and determinants of discrepancies of reported morbidity to actual morbidity. A table includes all determinants of discrepancies or differences of reported morbidity to actual morbidity identified during literature review and their belonging classes. (DOCX 25 kb)

Additional file 4: Appendix 3. Illustration of coding of each SIA item of cholera. Appendix 3 shows the assignment of code values of each item and the calculation the score for cholera.(DOCX 20 kb)

## Abbreviations

AFP: Acute flaccid paralysis
ARI: Acute respiratory infections
BD: Bloody diarrhoea
Cont.: Contagiousness
DD: Differential Diagnosis
DLN: Disease Local Name
DRC: Democratic Republic of Congo
DRT: Death rate (%) without Treatment
ECT: Etiological concept and type of disease
F&P Severity: Form and perceived severity
GDDC: General Direction of Disease Control
HF: Hemorrhagic fevers
IDSR: Integrated Disease Surveillance and Response
IFR: internationally funded research
IP: Incubation period
MNG: Meningitis
MPX: Monkeypox
NER: Number of epidemics reported each year during the last five years
NIEP: National/International eradication programs
NT: Neonatal tetanus
OD: Onset disease
PAE: Proportion of health zones affected by epidemics of each of the 15 diseases (%)
PPV: Positive predictive value
PRISMA: Preferred Reporting Items for Systematic reviews and Meta-Analyses
Response: Response Characteristic
SAP: Symptoms in the acute phase
SIA: Score of IDSR Adequacy
Spatial D: Spatial distribution of disease
TF: Typhoid fever
TR: Timely Response
WHO AFRO: WHO Regional Office for Africa
WHO: World Health Organization
YF: Yellow fever
