# Supplementary material for: The score of integrated disease surveillance and response adequacy (SIA): a pragmatic score for comparing weekly reported diseases based on a systematic review
Source: BMC Public Health. 2019 May 22;19:624. doi: 10.1186/s12889-019-6954-3 (PMC6532185; doi:10.1186/s12889-019-6954-3)
Supplement: Supplementary file 1 — Appendix 1. PRISMA 2009 Checklist. A checklist includes 27 items deemed essential for transparent reporting of a systematic review. (DOCX 32 kb). (DOCX 31 kb) [file 12889_2019_6954_MOESM1_ESM.docx]

| **Appendix 1: PRISMA 2009 Checklist** | | | |
| --- | --- | --- | --- |
| **Section/topic** | **#** | **Checklist item** | **Answers** |
| **TITLE** | | | |
| **Title** | 1 | Identify the report as a systematic review, meta-analysis, or both. | The tile includes the word ‘‘systematic review". |
| **ABSTRACT** | | | |
| Structured summary | 2 | Provide a structured summary including, as applicable: background; objectives; data sources; study eligibility criteria, participants, and interventions; study appraisal and synthesis methods; results; limitations; conclusions and implications of key findings; systematic review registration number. | The summary was written by including all these topics. |
| **INTRODUCTION** | | | |
| Rationale | 3 | Describe the rationale for the review in the context of what is already known. | The introduction was written rationally showing the context in which this review was conducted (see introduction). |
| Objectives | 4 | Provide an explicit statement of questions being addressed with reference to participants, interventions, comparisons, outcomes, and study design (PICOS). | The objective the subject of the study (IDSR reported data), the intervention (Assessing the level of adequacy), the comparator group (actual morbidity) and the outcome (Score of adequacy) were specified. |
| **METHODS** | | | |
| Protocol and registration | 5 | Indicate if a review protocol exists, if and where it can be accessed (e.g., Web address), and, if available, provide registration information including registration number. | We didn't elaborate a review protocol. However, databases of research, keywords and types of qualitative variables were predefined in advance with other members of the research team. |
| Eligibility criteria | 6 | Specify study characteristics (e.g., PICOS, length of follow-up) and report characteristics (e.g., years considered, language, publication status) used as criteria for eligibility, giving rationale. | A study was eligible if was founded one or some determinants of the discrepancies in the results of article and the mechanism of discrepancies explained in the discussion. Only English and French articles were included in the study. No publication date and no publication status restrictions were required. |
| Information sources | 7 | Describe all information sources (e.g., databases with dates of coverage, contact with study authors to identify additional studies) in the search and date last searched. | The systematic review was performed on seven databases (Embase, Medline, Web of Science, Cochrane, Scopus, Cairn.info and Persée) from their inception to December 2016. Technical guides and reports were identified through manual searches. |
| Search | 8 | Present full electronic search strategy for at least one database, including any limits used, such that it could be repeated. | The following Keywords were used in the English language: *epidemiological surveillance, administrative data, health information, health statistics, reported morbidity, real morbidity and data quality*. The same translated Keywords were used in the French language. Queries combining these words were then constructed using the Boolean search operator “AND”, “OR” to identify the most relevant articles. |
| Study selection | 9 | State the process for selecting studies (i.e., screening, eligibility, included in systematic review, and, if applicable, included in the meta-analysis). | The identified articles were screened independently by two reviewers on basis of title and abstract and applied the eligibility criteria to include them in the review. Disagreements between reviewers were resolved by consensus. |
| Data collection process | 10 | Describe method of data extraction from reports (e.g., piloted forms, independently, in duplicate) and any processes for obtaining and confirming data from investigators. | Data were extracted from a questionnaire searching for each article the names of the authors, the year of publication, the country of the study and determinants independently and in duplicate by two reviewers. Disagreements between reviewers were resolved by consensus. |
| Data items | 11 | List and define all variables for which data were sought (e.g., PICOS, funding sources) and any assumptions and simplifications made. | We identified 23 classes of determinants but only 12 determinants were used to construct the score according to the following criteria: availability, discriminatory ability, sensitivity and reproducibility. |
| Risk of bias in individual studies | 12 | Describe methods used for assessing risk of bias of individual studies (including specification of whether this was done at the study or outcome level), and how this information is to be used in any data synthesis. | The Risk of bias in individual studies was not assessed because we didn't interest in measuring the relation between studies but we were looked for qualitative variables that could influence the reported data). |
| Summary measures | 13 | State the principal summary measures (e.g., risk ratio, difference in means). | Non available because we used the qualitative data which could not be summarized. |
| Synthesis of results | 14 | Describe the methods of handling data and combining results of studies, if done, including measures of consistency (e.g., I^2^) for each meta-analysis. | The identified determinants or items were presented in a table according to the steps between perceived and reported morbidity/mortality. |
| Risk of bias across studies | 15 | Specify any assessment of risk of bias that may affect the cumulative evidence (e.g., publication bias, selective reporting within studies). | Technical guides and reports were identified using manual searches and results were incorporated. |
| Additional analyses | 16 | Describe methods of additional analyses (e.g., sensitivity or subgroup analyses, meta-regression), if done, indicating which were pre-specified. | To check the robustness of the score, a sensitivity analysis was performed by removing iteratively each item and by modifying the numerical values of the item codes. |
| **RESULTS** | | | |
| Study selection | 17 | Give numbers of studies screened, assessed for eligibility, and included in the review, with reasons for exclusions at each stage, ideally with a flow diagram. | Seven hundred fifty articles were screened on basis of title and abstract. Among those 450 were not selected because they were published in a language other than French or English, or their abstracts and/or full text were not available and or their contents were redundant to those already selected. Seventy-one articles and 35 other documents were selected matching the criteria of eligibility. The flow diagram was elaborate (see figure 1). |
| Study characteristics | 18 | For each study, present characteristics for which data were extracted (e.g., study size, PICOS, follow-up period) and provide the citations. | The selected studies were summarized in the results with the following characteristics: country of study, year and language of publication. |
| Risk of bias within studies | 19 | Present data on risk of bias of each study and, if available, any outcome level assessment (see item 12). | Not available. See point 12. |
| Results of individual studies | 20 | For all outcomes considered (benefits or harms), present, for each study: (a) simple summary data for each intervention group (b) effect estimates and confidence intervals, ideally with a forest plot. | Given the large number of articles selected (71), the results of the studies were summarized in the table 1 representing determinant of discrepancies , class of determinant and references according to the steps between perceived and reported morbidity/mortality. |
| Synthesis of results | 21 | Present results of each meta-analysis done, including confidence intervals and measures of consistency. | An adequacy score (Score of IDSR Adequacy or SIA) was constructed. It was composed by twelve items which were grouped in six dimensions. The SIA was applied to the 15 weekly reported diseases by the IDSR programme in DRC. The 15 IDSR diseases were classified in high, moderate or low adequacy group after using the Jenks discretization. |
| Risk of bias across studies | 22 | Present results of any assessment of risk of bias across studies (see Item 15). | Non available for the reasons mentioned in point 15. |
| Additional analysis | 23 | Give results of additional analyses, if done (e.g., sensitivity or subgroup analyses, meta-regression [see Item 16]). | The results obtained from the sensitivity analysis (iteratively removing the items and modifying the code values) are presented in Tables 4 and 5. |
|  | | | |
| Summary of evidence | 24 | Summarize the main findings including the strength of evidence for each main outcome; consider their relevance to key groups (e.g., healthcare providers, users, and policy makers). | Healthcare providers, searchers and policy maker could now use the SIA to assess adequacy of IDSR data. Depending on the high, moderate or low adequacy group the disease are allocated to. Disease information could not be considered at the same level. |
| Limitations | 25 | Discuss limitations at study and outcome level (e.g., risk of bias), and at review-level (e.g., incomplete retrieval of identified research, reporting bias). | The absence of a review protocol could manly affect the results of the study based on quantitative data. In addition, the definition in advance of databases of research, keywords and types of qualitative variables with other members of the research team well guided the collection of articles and variables. |
| Conclusions | 26 | Provide a general interpretation of the results in the context of other evidence, and implications for future research. | The SIA allow to classify reported IDSR data in 3 categories:” usable”, “usable after adjustment” or “non-usable. The SIA is a beneficial tool that could help to better classify, optimize and interpret data both in research and public health. |
| **FUNDING** | | | |
| Funding | 27 | Describe sources of funding for the systematic review and other support (e.g., supply of data); role of funders for the systematic review. | The project was funded by the PICS CNRS France 2015 with number System: 263320. However, no role was played by the funders during the systematic review. |
