# Supplementary material for: The score of integrated disease surveillance and response adequacy (SIA): a pragmatic score for comparing weekly reported diseases based on a systematic review
Source: BMC Public Health. 2019 May 22;19:624. doi: 10.1186/s12889-019-6954-3 (PMC6532185; doi:10.1186/s12889-019-6954-3)
Supplement: Supplementary file 2 — Appendix 2. . Database search strategies. Description of data: Appendix 2 includes the search terms for the epidemiological surveillance, administrative data, health information, health statistics, reported morbidity, real morbidity and data quality. (DOCX 35 kb) [file 12889_2019_6954_MOESM2_ESM.docx]

**Appendix 2: Database search strategies**

**THE COCHRANE LIBRARY**

#1 MeSH descriptor Epidemiologic surveillance explode all trees

#2 (IDSR in All Text or “Integrated Disease Surveillance” in All Text)

#3 (disease* in All Text near/5 surveillance in All Text)

#4 (epidem* in All Text near/5 monitor* in All Text)

#5 (health in All Text near/5 monitor* in All Text)

#6 (sanitary in All Text near/5 supervision? in All Text)

#7 (“IDSR” or “disease* surveillance” or “epidem* monitor*” or “health monitor*” or “sanitary supervision?” in All Text)

#8 (#1 or #2 or #3 or #4 or #5 or #6 or #7)

#9 MeSH descriptor Health Information Systems explode all trees

#10 (report* in All Text near/5 data in All Text)

#11 (diagnos* in All Text near/5 morbid* in All Text)

#12 (administrat* in All Text near/5 data in All Text)

#13 (health in All Text near/5 information in All Text)

#14 (health in All Text near/5 statistic* in All Text)

#15 (“report* data” or “diagnos* morbid*” or “administrat* data” or “health information” or “health statistic*” in All Text)

#16 (#9 or #10 or #11 or #12 or #13 or #14 or #15)

#17 (#8 or #16)

#18 MeSH descriptor morbidity explode all trees

#19 (actual in All Text or real in All Text near/5 morbid* in All Text)

#20 (#18 or #19)

#21 MeSH descriptor Data quality explode all trees

#22 (accurac* in All Text near/5 data in All Text)

#23 (“data quality” or “accurac* data” in All Text)

#24 (#21 or #22 or #23)

#25 (#17 and #20 and #24)

**MEDLINE**

#1 exp Epidemiologic surveillance/

#2 (IDSR or “Integrated Disease Surveillance”).tw,ot.

#3 (disease$ adj3 surveillance).tw,ot.

#4 (epidem$ adj3 monitor$).tw,ot.

#5 (health adj3 monitor$).tw,ot.

#6 (sanitary adj3 supervision?).tw,ot.

#7 (“disease$ surveillance” or “epidem$ monitor$” or “health monitor$” or “sanitary supervision?”).tw,ot.

#8 (#1 or #2 or #3 or #4 or #5 or #6 or #7)

#9 exp Health Information Systems/

#10 (report adj3 data).tw,ot.

#11 (diagnos$ adj3 morbid$).tw,ot.

#12 (administrat$ adj3 data).tw,ot.

#13 (health adj3 information).tw,ot.

#14 (health adj3 statistic$).tw,ot.

#15 (“report$ data” or “diagnos$ morbid$” or “administrat$ data” or “health information” or “health statistic$”).tw,ot.

#16 (#9 or #10 or #11 or #12 or #13 or #14 or #15)

#17 (#8 or #16)

#18 exp morbidity/

#19 (actual or real adj3 morbid$).tw,ot.

#20 (#18 or #19)

#21 exp Data quality/

#22 (accurac$ adj3 data).tw,ot.

#23 (“data quality” or “accurac$ data”).tw,ot.

#24 (#21 or #22 or #23)

#25 (#17 and #20 and #20)

**EMBASE**

#1 exp Epidemiologic surveillance/

#2 (IDSR or “Integrated Disease Surveillance”).tw,ot.

#3 (disease$ adj3 surveillance).tw,ot.

#4 (epidem$ adj3 monitor$).tw,ot.

#5 (health adj3 monitor$).tw,ot.

#6 (sanitary adj3 supervision?).tw,ot.

#7 (“disease$ surveillance” or “epidem$ monitor$” or “health monitor$” or “sanitary supervision?”).tw,ot.

#8 (#1 or #2 or #3 or #4 or #5 or #6 or #7)

#9 exp Health Information Systems/

#10 (report adj3 data).tw,ot.

#11 (diagnos$ adj3 morbid$).tw,ot.

#12 (administrat$ adj3 data).tw,ot.

#13 (health adj3 information).tw,ot.

#14 (health adj3 statistic$).tw,ot.

#15 (“report$ data” or “diagnos$ morbid$” or “administrat$ data” or “health information” or “health statistic$”).tw,ot.

#16 (#9 or #10 or #11 or #12 or #13 or #14 or #15)

#17 (#8 or #16)

#18 exp morbidity/

#19 (actual or real adj3 morbid$).tw,ot.

#20 (#18 or #19)

#21 exp Data quality/

#22 (accurac$ adj3 data).tw,ot.

#23 (“data quality” or “accurac$ data”).tw,ot.

#24 (#21 or #22 or #23)

#25 (#17 and #20 and #20)

**WEB OF SCIENCE**

#1 TS=(“IDSR” or “Integrated Disease Surveillance” or “disease* surveillance” or “epidem* monitor*” or “health monitor*” or “sanitary supervision?”)

#2 TS=(“report* data” or “diagnos* morbid*” or “administrat* data” or “health information” or “health statistic*”)

#3 (#1 or #2)

#4 TS=(“actual morbid*” or “real morbid*”)

#5 TS=(“data quality” or “accurac* data”)

#6 (#3 and #4 and #5)

**SCOPUS**

#1 TITLE-ABS-KEY (“IDSR” or “Integrated Disease Surveillance” or “Disease PRE/2 Surveillance” or “Disease W/2 Surveillance” or “disease* surveillance” or “epidem* monitor*” or “health monitor*” or “sanitary supervision?”)

#2 TITLE-ABS-KEY (“report* data” or “diagnos* morbid*” or “administrat* data” or “health information” or “health statistic*”)

#3 (#1 or #2)

#4 TITLE-ABS-KEY (“actual morbid*” or “real morbid*”)

#5 TITLE-ABS-KEY (“data quality” or “accurac* data”)

#6 (#3 and #4 and #5)

**PERSEE**

#1 («SIMR » OU «Surveillance intégrée des maladies» OU «surveillance épidémiologique» OU «veille sanitaire»)

#2 («données administratives» OU «informations sanitaires» OU «statistiques sanitaires» OU «morbidité rapportée» OU «morbidité diagnostiquée»)

#3 (#1 OU #2)

#4 («morbidité réelle»)

#5 («qualité de données»)

#6 (#3 ET #4 ET #5)

#7 Limités #6 aux langues=français et anglais, aux années=1998 à 2017 et aux collections=anthropologie et sociétés, sciences sociales et santé.

**CAIRN.INFO**

#1 (SIMR  OU Surveillance intégrée des maladies OU surveillance épidémiologique OU «veille sanitaire)

#2 (données administratives OU informations sanitaires OU statistiques sanitaires OU morbidité rapportée OU morbidité diagnostiquée)

#3 (#1 OU #2)

#4 (morbidité réelle)

#5 (qualité de données)

#6 (#3 ET #4 ET #5)

#7 Limités #6 aux disciplines=sociologie et sociétés, médecine et Santé publique.
