# Supplementary material for: The score of integrated disease surveillance and response adequacy (SIA): a pragmatic score for comparing weekly reported diseases based on a systematic review
Source: BMC Public Health. 2019 May 22;19:624. doi: 10.1186/s12889-019-6954-3 (PMC6532185; doi:10.1186/s12889-019-6954-3)
Supplement: Supplementary file 3 — Table S1. Classes and determinants of discrepancies of reported morbidity to actual morbidity. A table includes all determinants of discrepancies or differences of reported morbidity to actual morbidity identified during literature review and their belonging classes. (DOCX 25 kb) [file 12889_2019_6954_MOESM3_ESM.docx]

**Table S1**. Determinants of discrepancies of reported morbidity to actual morbidity and their classes.

| Course of patients | Determinants or potential distorting factors in their classes | References |
| --- | --- | --- |
| Perception of the illness and  Health care access | **Form and Perceived severity of illness** (e.g.: chronic/acute, sever/mild, onset, acute phase, incubation period, infectiosity, fatality, etc.) | [24-29] |
|  | **Etiological concept and type of disease** (e.g.: popular or religious beliefs, disease local name, community diagnosis of the disease, cultural barriers between patients and caregivers…) | [27, 29, 30-36] |
|  | Individual characteristics of patients (e.g.: age, sex, gender, etc.) | [27, 32, 37-44] |
|  | Socio-economic characteristics of patients (e.g.: education, income, socio-economic status and occupation, household size and status in household, interactions with social network, ethnic group and religion...) | [27, 32, 43-54] |
|  | Attractiveness factors of places (e.g.: markets, roads, railway, administrative headquarters, etc. | [55-60] |
|  | Geographical accessibility (e.g.: distance of health structures from patients residences) | [27, 31-33, 50, 61-64] |
|  | Characteristics of the environment (e.g.: forests, relief, climate, hydrographic, road, etc.) | [55, 65-67] |
|  | Urbanization (e.g.: inequalities in the care of different parts of a city) | [68-72] |
|  | Conjuncture (e.g.: fishing, hunting, etc.) movements of population (eg.: conflicts, famine, etc.) | [73] |
| Diagnosis | Presence of an intervention program (e.g.: active surveillance program, etc.) | [19, 74, 75] |
|  | Functioning of health services: quality of service and framework (e.g.: reduction of wait times, continuity in staff, rehabilitated and equipped services,...) | [27, 33, 37, 47, 51, 76, 78-80] |
|  | Staff competence (e.g.: qualification, training, etc.) | [33, 51, 76, 77] |
|  | Supplementary diagnosis (biology, etc.) | [33, 81] |
|  | Clinical standardized decision tree for diagnosing (e.g.: flowcharts, etc.) | [77, 82, 83] |
|  | **Difficulty of differential diagnosis with other diseases** (e.g.: annual notification of outbreaks, reproductivity, validity or positive predictive value, etc.) | [16, 84-87] |
|  | **Spatial distribution of disease** (e.g.: distribution area of outbreaks, etc.) | [88-90] |
| Data reporting | Staff competence (e.g.: qualification, training, supervision, etc.) | [73] |
|  | Standardization of data collection tools (same data collection tools everywhere, guidelines for collection, centralization and reporting of data, etc.) | [73, 91] |
|  | Large volume of work (non-compliance of guidelines for collection, centralization and reporting of data, etc....). | [84] |
|  | Falsification of data (to increase bonus, fear of hierarchy, etc.) | [84] |
|  | Typing errors (lack of attention, a large work volume, etc.) | [84, 93] |
|  | **Integration of the disease into a national or global strategy** (financial support by a partner, elimination or eradication program, global strategy, etc.) | [10, 92] |
|  | **Characteristic of the response** (immediate response or not, etc.) | [10, 92] |
