# Supplementary material for: The score of integrated disease surveillance and response adequacy (SIA): a pragmatic score for comparing weekly reported diseases based on a systematic review
Source: BMC Public Health. 2019 May 22;19:624. doi: 10.1186/s12889-019-6954-3 (PMC6532185; doi:10.1186/s12889-019-6954-3)
Supplement: Supplementary file 4 — Appendix 3. Illustration of coding of each SIA item of cholera. Appendix 3 shows the assignment of code values of each item and the calculation the score for cholera.(DOCX 20 kb) [file 12889_2019_6954_MOESM4_ESM.docx]

**Appendix 3: Illustration of coding of each SIA item of cholera.**

Twelve determinants (which became items of the score according to the scaling terminology) were selected and summarized in Table 2. For each disease, the response of each item of the SIA was coded as 0/1, 0/2 or 0/1/2. For instance, the items of cholera were coded as follows:

- Incubation period (IP) was coded 2, because its IP is few hours to 5 days. This corresponds to code 2 of the item, which is less than 7 days.
- Onset (OD) was coded 2, because its SMO is severe with acute symptoms (diarrhea with large dehydration). This corresponds to code 2 of the item, which is severe with symptoms.
- Symptoms in the acute phase (SAP) was coded 2, because its APS is severe with diarrhea. This corresponds to code 2 of the item, which is Severe.
- Contagiousness (Cont) was coded 2, because cholera is a very contagious disease. This corresponds to code 2 of the item, which is high contagiousness.
- Death rate (%) without Treatment (DRT) was coded 2, because cholera has a high case-fatality (25 à 50 %). This corresponds to code 2 of the item, which is greater than or equal to 20%.
- Disease Local Name (DLN) was coded 2, because cholera has a disease local name shared by localities (i.e. well known by the populations). This corresponds to code 2 of the item, which is shared by localities.
- Number of epidemics reported each year during the last five years (NER) was coded 2, because during this period, five or more outbreaks of cholera occurred in DRC. This corresponds to code 2 of the item, which is equal to five.
- Positive predictive value (PPV) was coded 1, because its PPV was 33 %. This number corresponds to code 1 of the item, which takes values between 20 and 50 %.
- Proportion of health zones affected by epidemics of each of the 15 diseases (%) (PAE) was coded 1, because more than 20% of health zones of DRC reported outbreaks of cholera. This corresponds to code 2 of the item, which is greater than or equal to 20%.
- internationally funded research (IFR) was coded 1, because cholera has an international financial support in DRC. This corresponds to code 1 of the item, which is with support.
- National/International eradication programs (NIEP) was coded 1, because cholera is part of an elimination program in DRC. This corresponds to code 1 of the item, which is yes.
- Timely Response (TR) was coded 1, because cholera is because cholera is part of immediate-notification diseases in DRC. This corresponds to code 1 of the item, which is immediate response.

The sum of all code values of cholera was 19 (Please see Table 3).
